# Identification of a novel natural compound inhibitor targeting AmpC β-lactamase to combat multidrug-resistant *Pseudomonas aeruginosa*

**DOI:** 10.3389/fmicb.2026.1813201

**Published:** 2026-05-19

**Authors:** Santhosh Mudipalli Elavarasu, Sasikumar K

**Affiliations:** 1 Department of Integrative Biology, School of Biosciences and Technology, Vellore Institute of Technology (VIT), Vellore, India; 2 Department of Sensor and Biomedical Technology, School of Electronics Engineering, Vellore Institute of Technology (VIT), Vellore, India

**Keywords:** AmpC β-lactamase, antimicrobial resistance, molecular dynamics, *Pseudomonas aeruginosa*, virtual screening

## Abstract

**Introduction:**

Antimicrobial resistance (AMR) remains one of the most urgent threats to global public health, driven by the rising prevalence of multidrug-resistant (MDR) pathogens such as Pseudomonas aeruginosa. A key mediator of resistance in this organism is AmpC β-lactamase, an enzyme that confers bacterial survival by hydrolysing β-lactam antibiotics before they can exert their effect.

**Methods:**

We screened 374 compounds from the ChemDiv chemical library for novel inhibitors that target this enzyme, with a bias toward natural-compound-derived scaffolds as an alternative to existing resistance-countering strategies. Pharmacokinetic profiling was performed using SwissADME. Molecular dynamics (MD) simulations were carried out to further characterize the stability of the protein-ligand complex. Additionally, free energy perturbation (FEP) calculations were performed to quantitatively verify the binding affinity towards AmpC β-lactamase.

**Results:**

Among the screened compounds, compound N094-0017 emerged as the top candidate. It showed good affinity for the AmpC active site and formed stable hydrogen bonds, with a binding energy of −5.6 kcal/mol. SwissADME-based pharmacokinetic profiling suggested desirable drug-like and favourable oral bioavailability profiles. MD simulations were conducted to further characterize the stability of the protein-ligand complex, and the RMSD, RMSF, and SASA profiles were consistent, confirming stable retention of the ligand within the active site throughout the simulation period. Moreover, FEP calculations showed a binding free energy of ∼40 kJ/mol for N094-0017 toward AmpC β-lactamase.

**Discussion:**

In conclusion, these data suggest N094-0017 as a promising candidate for an AmpC β-lactamase inhibitor, and natural compounds present a promising strategy for addressing the growing challenge of AMR.

## Graphical Abstract

**Figure:**
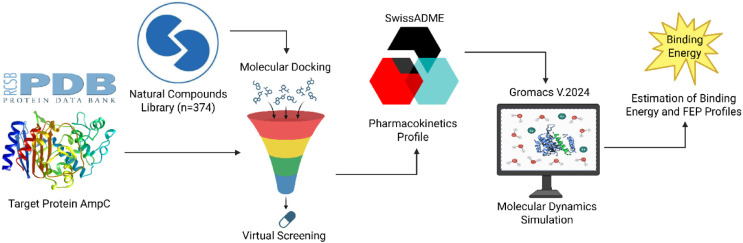
Computational workflow for the identification and evaluation of potential inhibitors targeting AmpC β-lactamase.

## 1 Introduction

A major global health concern is antimicrobial resistance (AMR), as multidrug-resistant (MDR) bacteria cause significant morbidity and mortality worldwide (Murray et al., 2022). Non-fermenting Gram-negative bacteria are particularly concerning contributors to AMR, especially in nosocomial infections among critically ill and immunocompromised patients (Sadyrbaeva-Dolgova et al., 2023; Mukomena et al., 2024). A major source of serious infections, such as bloodstream infections, surgical site infections, and pneumonia, *Pseudomonas aeruginosa* (*P. aeruginosa*) disproportionately affects vulnerable groups such as patients with cystic fibrosis, burn victims, and intensive care unit patients. These infections contribute significantly to global morbidity and mortality (Recio et al., 2020; Sendra et al., 2024). Antibiotic-resistant bacterial infections are estimated to cause nearly 700,000 deaths annually worldwide, with *P. aeruginosa* recognized as an important opportunistic pathogen contributing to this burden (Qin et al., 2022). The rising prevalence of multidrug-resistant (MDR) strains of *P. aeruginosa* has further complicated treatment strategies, highlighting the need to better understand its mechanisms of resistance and to develop more effective therapeutic interventions (Pang et al., 2019). This pathogen has evolved several overlapping resistance strategies that collectively make it exceptionally difficult to treat. These include acquired resistance through mutations and horizontal gene transfer, adaptive resistance mediated by biofilm formation and persister cell development, and intrinsic resistance arising from poor outer-membrane permeability (Angus et al., 1982; Okamoto et al., 2001; Poole, 2011; Thi et al., 2020; Langendonk et al., 2021; Zhao et al., 2024; Lorusso et al., 2022). Together, these mechanisms allow *P. aeruginosa* to evade a broad range of commonly used antibiotics, including aminoglycosides, fluoroquinolones, and β-lactams (Giovagnorio et al., 2023). Among the various resistance mechanisms, AmpC β-lactamase production is particularly significant, as it directly undermines the activity of several β-lactam antibiotics, including penicillins, cephalosporins, and monobactams (Kong et al., 2005). AmpC β-lactamases are widely produced among Gram-negative bacteria and work by hydrolyzing these antibiotics, stripping them of their antimicrobial activity before they can reach their target (Drawz et al., 2011; Philippon et al., 2022). Over time, this enzymatic activity, driven largely by selective antibiotic pressure, has contributed to the emergence of multidrug and even pan-drug resistance phenotypes. The situation is made worse by mutations in genes involved in cell wall recycling and peptidoglycan metabolism, particularly *ampD* and *dacB*. Mutations in *ampD*, which normally regulates muropeptide recycling, and in *dacB*, which encodes the penicillin-binding protein PBP4, represent two well-characterized pathways that lead to stable AmpC hyperproduction and heightened β-lactamase activity in *P. aeruginosa* (Beadle et al., 1999; Pérez-Gallego et al., 2016; Glen and Lamont, 2021; Cabot et al., 2014). In clinical practice, AmpC-mediated resistance is typically managed using β-lactam/β-lactamase inhibitor (BLBLI) combinations. Ceftazidime/avibactam and ceftolozane/tazobactam have both demonstrated activity against AmpC-producing isolates (Yahav et al., 2020). However, resistance driven by *ampC* overexpression and structural mutations has begun to erode their usefulness (Berrazeg et al., 2015). Treatment plans are complicated by the fact that these inhibitors are ineffective against strains that produce metallo-β-lactamases. Imipenem/relebactam has shown promise against certain resistant strains, but its efficacy can be compromised by mechanisms such as porin loss, efflux pump overexpression, and carbapenemase production (Heo, 2021). The problem is further compounded in biofilm-positive strains of *P. aeruginosa*, where AmpC production is significantly elevated (Heydari and Eftekhar, 2015). Biofilms act as protective barriers, reducing antibiotic penetration and promoting the overexpression of resistance mechanisms, complicating treatment outcomes (Mirghani et al., 2022). The high AmpC levels seen in biofilm-associated strains greatly reduce the effectiveness of existing β-lactam therapies, leaving clinicians with few reliable options. Taken together, these challenges make a compelling case for new and more targeted therapeutic approaches to address AmpC-mediated resistance in *P. aeruginosa*. In this regard, bioinformatics-driven strategies have gained considerable traction as a practical route toward identifying novel inhibitors. Rather than focusing solely on developing new antibiotics, these computational approaches aim to design and optimize natural compounds that directly disable resistance mechanisms. Through virtual screening, molecular docking, and simulation-based analyses, it is now possible to systematically identify candidates that can effectively block AmpC β-lactamase activity. Such natural-compound-based inhibitors hold real promise as adjuncts to existing β-lactam antibiotics, potentially restoring their efficacy and rendering resistant *P. aeruginosa* strains susceptible once again.

## 2 Materials and methods

### 2.1 Ligand library and preparation

For virtual screening, we selected the ChemDiv Natural Compound Library, a curated collection of 374 structurally diverse natural compounds with lead-like and drug-like characteristics. This library was chosen because it brings together chemically varied scaffolds with well-established pharmacological relevance and favorable drug-like properties, making it particularly well-suited for focused screening efforts against bacterial resistance targets. The compounds within this library span a wide range of biological origins, including plants, microorganisms, and marine organisms, all of which have long been recognized as rich sources of pharmacologically active molecules. The ligands were collected in Structure Data File (SDF) format and processed with the Schrödinger Suite (Schrödinger, LLC, New York, United States) (Lipinski, 2004). The LigPrep module was used to prepare the ligands and construct energetically optimal three-dimensional (3D) conformations. The Epik module was used to generate ionization states at physiological pH (7.0 ± 2.0), with salts removed as needed. To ensure proper conformational sampling, stereochemical variations were examined, with up to 32 stereoisomers generated for each drug. To acquire low-energy ligand conformations appropriate for docking analysis, energy minimization was conducted using the OPLS3 force field and default parameters.

### 2.2 Protein preparation

The X-ray crystal structure of AmpC β-lactamase from *P. aeruginosa* was obtained from the RCSB Protein Data Bank (PDB ID: 6DPT). The Protein Preparation Wizard from the Schrödinger Suite was used to prepare the protein. The structure was pre-processed by assigning bond ordering, introducing hydrogen atoms, defining disulfide bonds, and eliminating non-essential heteroatoms and water molecules. The hydrogen-bonding network was then carefully optimized, and restrained energy minimization was performed using the OPLS3 force field with a default RMSD cutoff of 0.30 Å. This step was important for relieving steric clashes within the structure while keeping the overall protein conformation intact (Harder et al., 2016). Potential binding pockets on the AmpC β-lactamase surface were subsequently identified using CASTpFold, after which a receptor grid was constructed around the active site using the Receptor Grid Generation module. This grid was defined to encompass the catalytic residues directly responsible for β-lactam hydrolysis, ensuring that the docking calculations were appropriately focused on the functionally relevant region of the enzyme (Ye et al., 2024).

### 2.3 Molecular docking and virtual screening

To assess how well each ligand could bind to the AmpC β-lactamase active site, molecular docking was carried out using the Glide module within the Schrödinger suite (Friesner et al., 2004). The process followed a stepwise screening approach to progressively narrow down the most promising candidates. In the first stage, all prepared compounds were screened using High-Throughput Virtual Screening (HTVS), enabling rapid filtering based on preliminary docking scores and interaction patterns. Compounds that performed well in this initial screen were then taken forward for Standard Precision (SP) docking, offering a more refined evaluation of binding behavior (Friesner et al., 2006). The top-ranking compounds from SP docking were subsequently subjected to Extra Precision (XP) docking, which provided the most rigorous assessment of binding poses. During XP docking, van der Waals radii were scaled at a factor of 0.80, and a partial charge cutoff of 0.15 was applied to improve the accuracy of pose discrimination. Following docking, energy minimization was performed on the resulting complexes, and the nature of protein–ligand interactions was examined using the Maestro visualization interface. Ligands were ranked by docking scores (expressed as ΔG in kcal/mol), and those with the highest binding affinity were shortlisted for further investigation via molecular dynamics simulations and free-energy calculations.

### 2.4 Pharmacokinetic evaluation

To assess whether the top four docking hits could realistically function as drug candidates, their pharmacokinetic and drug-likeness profiles were examined using the SwissADME web server (Daina et al., 2017). The canonical SMILES strings of each selected compound were submitted to the platform, which predicted a range of ADME-related properties. These included lipophilicity, aqueous solubility, gastrointestinal absorption, blood–brain barrier permeability, and potential interactions with cytochrome P450 enzymes, factors that collectively determine how a compound is likely to behave within a biological system. Drug-likeness was evaluated against several well-established screening criteria, including Lipinski’s rule of five, along with the Ghose, Veber, Egan, and Muegge filters. Together, these parameters provided a comprehensive picture of each compound’s oral bioavailability and overall suitability as a therapeutic agent. Compounds that demonstrated favorable profiles across these assessments were considered strong candidates for progression into molecular dynamics simulations and, ultimately, experimental validation.

### 2.5 MD simulation analysis

Molecular dynamics (MD) simulations were carried out using GROMACS version 2024, with the AmpC-ligand complexes showing the most favorable binding interactions and highest docking scores selected for a 500 ns simulation run. System input files were prepared through the CHARMM-GUI solution builder, and the CHARMM36 force field was applied for parameterization (Guterres et al., 2022). Each complex was placed within a cubic simulation box solvated with TIP3P water molecules, maintaining a 10 Å buffer between the protein surface and the box edges, after which counterions were introduced to achieve overall charge neutrality (Jorgensen et al., 1983). Non-bonded interactions, covering both van der Waals forces and short-range electrostatics, were handled using the Verlet cutoff scheme with a cutoff distance of 10 Å, while long-range electrostatic interactions were treated with the particle mesh Ewald (PME) method to ensure computational accuracy. Bond lengths were constrained throughout the simulation using the LINCS algorithm to preserve system integrity (Hess et al., 1997). Prior to the production run, the system underwent energy minimization using the steepest descent method to resolve any steric clashes and bring the system to a stable starting configuration (Van Der Spoel et al., 2005). Equilibration was then carried out in two successive phases, first under the NVT ensemble to stabilize temperature at constant volume, followed by the NPT ensemble to equilibrate both pressure and temperature. Temperature control during NPT equilibration was maintained using a thermostat, while a barostat ensured consistent pressure throughout (Bussi et al., 2007). Production simulations were run with a 2 fs integration time step, with coordinates saved every 1 ps to enable detailed trajectory analysis (Nesabi et al., 2024). In addition, the CHARMM-GUI platform provided a Python script that ensured GROMACS compatibility by easily converting topology (.top) and parameter (.itp) files. During the production phase, periodic boundary conditions and carefully selected parameters enabled the development of a robust and accurate simulation framework for analyzing the binding stability and dynamics of AmpC-ligand complexes. The trajectories were analyzed using GROMACS’s gmx analysis tools, with a focus on root-mean-square deviation (RMSD) to track structural variations, root-mean-square fluctuation (RMSF) to measure residue flexibility, the radius of gyration (Rg) to assess protein compactness during simulation, and other analyses.

### 2.6 PCA analysis in molecular dynamics

To perform principal component analysis (PCA), the GROMACS software suite, along with the gmx anaeig and gmx covar tools, were utilized. Since Cα atoms in proteins are considered reliable indicators of major conformational changes, they were the focus of the analysis. Based on the Cα atom positions during the simulation, the covariance matrix was first computed (Amadei et al., 1993; David and Jacobs, 2014). This matrix was then diagonalized to determine its eigenvalues and eigenvectors. The eigenvalues measure the amplitude of the protein’s motion, whereas the eigenvectors indicate the direction of these motions. The principal components (PCs) were determined by projecting the eigenvectors onto the protein’s coordinates, thereby identifying the most significant motions within the system. These PCs elucidate the dominant modes of protein movement. The gmx analysis tools facilitated the visualization of the eigenvalues and eigenvectors, offering insights into the contribution of each motion mode to the overall dynamic behavior of the protein. All PCA analyses were performed using GROMACS version 2024 (Papaleo et al., 2009), providing a comprehensive suite of trajectory analysis and data visualization tools.

### 2.7 Free energy landscape analysis

To investigate the intrinsic structural changes occurring within the protein over the course of the simulation, a conformational sampling approach was adopted, using structural deviation and protein gyration as the primary reaction coordinates. The Free Energy Landscape (FEL) was computed using the gmx sham tool available within GROMACS, which utilized these two coordinates as the basis for calculation. By examining the joint probability distributions of structural deviation and gyration [ρ(p1, p2)], the relative probabilities of different conformational states adopted by the system were determined. The Gibbs free energy (ΔG) associated with each state was then calculated based on the simulation temperature (T) and the Boltzmann constant (kB). The resulting 2D FEL offered a detailed view of the protein’s structural dynamics throughout the simulation, mapping free energy variations along both reaction coordinates and highlighting regions of stability and transition. This representation allowed for a clearer understanding of how the protein explored its conformational space and which states were energetically favored. The use of GROMACS-based tools simplified the overall free energy calculation process while providing meaningful visual insight into the relative stability of the various protein conformations encountered during the simulation (Papaleo et al., 2009). To further improve the clarity and interpretability of the conformational data, the 2D FEL was subsequently converted into a three-dimensional representation using Python scripting (Python Software Foundation, 2019), enabling a more intuitive visualization of the energy surface and the conformational transitions taking place across the simulation trajectory.

### 2.8 Dynamic cross-correlation studies

To explore how different residues of AmpC move in relation to one another during the simulation, Dynamic Cross-Correlation Matrix (DCCM) analysis was conducted. Positional fluctuations of Cα atoms were extracted from the simulation trajectory, and the mean structure was subtracted to isolate time-dependent atomic displacements. Cross-correlation coefficients were then derived by normalizing the covariance of these fluctuations, producing values that range between −1 and +1. Positive values reflect residues moving in a correlated, cooperative manner, while negative values indicate anticorrelated motions where residues move in opposing directions. The resulting DCCM maps were generated using custom Python scripts and provided a residue-level picture of coordinated dynamics across the protein (Python Software Foundation, 2019; Yu and Dalby, 2020). These maps were especially helpful in identifying regions of coordinated movement and exposing long-range dynamic interactions within the AmpC structure, providing information that exceeds what standard flexibility analyses can deliver and highlighting how motion is transferred across different parts of the protein during ligand binding.

### 2.9 MM/PBSA

The binding free energy of the protein-ligand complexes was estimated using the Molecular Mechanics Poisson-Boltzmann Surface Area (MM-PBSA) method (Genheden and Ryde, 2015), a well-established method for determining binding free energies. This computation was carried out using the following (Equation 1):


Δ⁢G⁢b⁢i⁢n⁢d⁢i⁢n⁢g=Δ⁢E⁢m⁢m+Δ⁢G⁢p⁢o⁢l+Δ⁢G⁢n⁢p-T⁢Δ⁢S (1)

The binding free energy (ΔGbinding), polar solvation energy (ΔGpol), non-polar solvation energy (ΔGnp), and total molecular mechanics energy in the gas phase (ΔEmm), which includes both bound and non-bonded interactions. The conformational entropy at temperature (T) is represented by (TΔS). To decrease computational expenses, we eliminated the entropy contribution (TΔS), assuming insignificant entropy changes among comparable ampC-ligand systems. This simplification, however, may impair accuracy if major structural shifts occur. Under this assumption, the binding free energy equation is written as follows (Equation 2):


Δ⁢G⁢b⁢i⁢n⁢d⁢i⁢n⁢g=Δ⁢E⁢b⁢o⁢n⁢d⁢e⁢d+Δ⁢E⁢n⁢o⁢n-b⁢o⁢n⁢d⁢e⁢d+Δ⁢G⁢p⁢o⁢l+Δ⁢G⁢n⁢p-T⁢Δ⁢S (2)

The single-trajectory method assumes that the structures of the bound and unbound protein-ligand complexes are identical, which leads to ΔEbonded = 0 (Homeyer and Gohlke, 2012). As a result, the equation simplifies further (Equation 3):


Δ⁢G⁢b⁢i⁢n⁢d⁢i⁢n⁢g=Δ⁢E⁢v⁢d⁢w+Δ⁢E⁢e⁢l⁢e+Δ⁢G⁢p⁢o⁢l+Δ⁢G⁢n⁢p-T⁢Δ⁢S (3)

In this context, Van der Waals interactions are denoted as ΔEvdw, while electrostatic interactions are represented as ΔEele. The binding free energy components, excluding the entropy term, were calculated with the g_mmpbsa program (Kumari et al., 2014). A total of 1,000 snapshots, taken at 20 ps intervals from the last 20 ns of the MD simulation, were used for this analysis. These snapshots enabled the estimation of the binding free energy of the AmpC-ligand complexes, offering a comprehensive view of their interaction energetics.

### 2.10 Alchemical free energy perturbation (FEP) calculations

The GROMACS molecular dynamics tool was used to run alchemical free energy perturbation (FEP) simulations and measure the absolute binding free energy of the ligand-AmpC β-lactamase complex (Van Der Spoel et al., 2005). The starting structure for the FEP calculations was obtained from the equilibrated protein–ligand complex after the molecular dynamics simulation. The ligand was alchemically decoupled from the system through a series of non-physical intermediate states defined by a coupling parameter (λ). The alchemical transformation was divided into 41 discrete λ windows, with electrostatic interactions of the ligand annihilated first over 21 windows (λ = 0.0–1.0), followed by van der Waals interaction decoupling over the remaining 21 windows. To avoid numerical instabilities associated with particle annihilation, a soft-core potential was applied to Lennard–Jones interactions using sc-alpha = 0.5 and sc-power = 1 (Beutler et al., 1994). The ligand was defined as a separate molecule type (UNK) in the system topology file.

Each λ window was simulated independently for 1 ns under isothermal–isobaric (NPT) conditions. Temperature throughout the simulation was held constant at 310 K using a stochastic dynamics integrator, while pressure was maintained at 1.0 bar using the Parrinello–Rahman barostat with isotropic pressure coupling (Parrinello and Rahman, 1981). A 2 fs time step was used for integration, and all hydrogen-bonded interactions were constrained by applying the LINCS algorithm to maintain structural stability during the run (Hess et al., 1997). Long-range electrostatic interactions were handled using the Particle Mesh Ewald (PME) method (Darden et al., 1993), and a uniform cutoff of 1.0 nm was applied for both short-range electrostatic and van der Waals interactions. Free energy differences between neighboring λ windows were estimated using the Bennett Acceptance Ratio (BAR) method, as implemented within the gmx bar tool in GROMACS (Bennett, 1976). To ensure that only well-equilibrated data contributed to the final estimates, the first 100 ps of each λ-window trajectory were discarded prior to analysis. The total binding free energy for each complex was then obtained by summing the individual free-energy contributions across all λ windows, providing a thermodynamically grounded assessment of ligand-binding affinity.

## 3 Results

### 3.1 Virtual screening

A structure-based virtual screening strategy was employed to identify potential inhibitors of AmpC β-lactamase. A total of 374 natural compounds from the ChemDiv database were screened against the active site of AmpC β-lactamase using the Glide docking module (Supplementary Figure 1). During ligand preparation, the initial compounds were expanded to 1,528 structures through the generation of stereoisomers and ionization states. After applying Lipinski filtering, 780 structures were retained for docking. These compounds were initially screened using High-Throughput Virtual Screening (HTVS), from which the top 16 unique compounds were selected. The shortlisted compounds were subsequently re-docked using the Standard Precision (SP) protocol, with all 16 compounds progressing to the next stage. Extra Precision (XP) docking was then performed for further refinement of binding interactions. Although 41 docking poses were generated at this stage, these corresponded to different binding conformations of the same 16 compounds. Based on docking scores and interaction profiles, five compounds (N094-0017, 0115-0006, N002-0028, N003-0017, and 0080-0056) were identified as the most promising candidates for further analysis. The synthesized ligands were docked into the receptor grid created around the active site, and their binding affinities were determined by Glide docking scores and interaction profiles. The hydrogen-bonding patterns and binding energies of the resulting protein-ligand complexes were investigated to assess binding stability and inhibitory potential (Table 1).

**TABLE 1:** Summarizes the docking analysis of compounds against the AmpC target protein.

AmpC protein with ligand complex	Binding affinity (kcal/mol)	H-bond	Interactive residues
Avibactam	−4.0	3	GLN120, ASN152, SER64
Ceftazidime	−3.9	4	ASN289, GLN120, ASN152, SER64
N094-0017	−5.6	2	HIS314, GLU272
0115-0006	−4.8	3	ASN289, ASN346, THR316
N002-0028	−4.8	3	ASN346, SER64, ALA318
N003-0017	−4.5	3	ASN152, ALA318, GLN120
0080-0056	−4.5	1	ASP264

**FIGURE 1:**
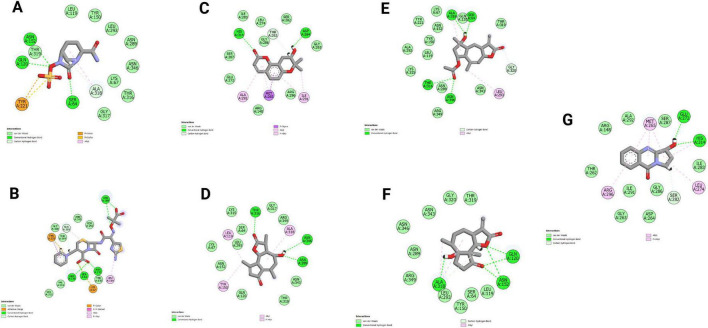
Illustrates the 2D interactions between the AmpC protein and various ligands. **(A)** Avibactam, **(B)** Ceftazidime, **(C)** 0080-0056, **(D)** 0115-0006, **(E)** N002-0028, **(F)** N003-0017, **(G)** N094-0017. Key binding features and interactions are highlighted.

Among all the compounds evaluated through virtual screening, the reference antibiotic ceftazidime recorded a Glide docking score of −3.9 kcal/mol and formed four hydrogen bonds with key active-site residues, namely GLN A:120, ASN A:152, and ASN A:289. The reference inhibitor, avibactam, yielded a comparable score of −4.0 kcal/mol and formed three hydrogen-bond interactions with SER A:64, GLN A:120, and ASN A:152. Among the test compounds, N094-0017 stood out with the highest binding affinity of all screened ligands, achieving a docking score of −5.6 kcal/mol and forming stable hydrogen bond interactions with GLU A:272 and HIS A:314 within the active site. Compound 0115-0006 recorded a docking score of −4.8 kcal/mol, with three hydrogen bonds observed at ASN A:289, THR A:316, and ASN A:346, while N002-0028 achieved an identical score of −4.8 kcal/mol through interactions with SER A:64, THR A:316, and ASN A:346. Compound N003-0017 scored −4.5 kcal/mol and engaged with residues GLN A:120, ASN A:152, and ALA A:318 via hydrogen bonding, and compound 0080-0056 achieved the same score of −4.5 kcal/mol while forming two hydrogen bonds with ASP A:264 and HIS A:314. Taken together, the virtual screening results revealed distinct binding modes and varied interaction profiles across the screened compound set. The notably good docking score and well-defined interaction network of N094-0017 point to a particularly favorable complementarity with the AmpC active site. Two-dimensional interaction diagrams for the top-ranked complexes are presented in Figure 1, and three-dimensional binding conformations are illustrated in Supplementary Figure 2. Based on these findings, N094-0017 emerges as the most promising candidate for progression to molecular dynamics simulations and binding free energy calculations.

### 3.2 Pharmacokinetic evaluation of the top compounds

To evaluate the drug-like potential of the top five compounds identified through virtual screening, a SwissADME analysis was performed covering their physicochemical properties, solubility, lipophilicity, pharmacokinetic behavior, and compliance with standard drug-likeness criteria. The detailed results are summarized in Tables 2, 3. All five ligands had molecular weights within the accepted range for orally bioavailable drug candidates, ranging from 202.21 to 306.35 Da. In terms of molecular flexibility, the compounds showed favorable profiles, with hydrogen-bond acceptors (HBAs) ranging from 3 to 5, hydrogen-bond donors (HBDs) of 1 across all compounds, and rotatable bond counts of 0 or 2. The topological polar surface area (TPSA) values ranged between 55.12 and 72.83 Å^2^, a range generally associated with good oral bioavailability. Aqueous solubility ranged from soluble to very soluble, and none of the compounds violated Lipinski’s rule of five, reinforcing their suitability as oral drug candidates. Lipophilicity assessment using both iLOGP and consensus LogP values indicated moderate hydrophobicity across the series, with iLOGP values ranging from 1.67 to 2.67. All ligands consistently achieved a bioavailability score of 0.55, demonstrated strong gastrointestinal absorption, and returned no PAINS alerts, suggesting a low likelihood of non-specific assay interference. Synthetic accessibility scores ranged from 2.75 to 4.91, indicating that all compounds are reasonably feasible to synthesize. Collectively, these results confirm that the selected ligands carry well-balanced drug-like characteristics, making them viable candidates for further structural optimization and experimental follow-up.

**TABLE 2:** Provides the physicochemical properties of the five highest-scoring compounds, including molecular weight (MW), hydrogen bond acceptors (H-BA), hydrogen bond donors (H-BD), and rotatable bonds (rBonds).

Compounds	Structure	MW	H-BA	H-BD	rBonds
N094-0017	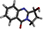	202.21	3	1	0
0115_0006	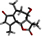	262.3	4	1	0
N002-0028	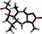	306.35	5	1	2
N003-0017	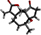	264.32	4	1	0
0080-0056	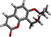	246.26	4	1	0

**TABLE 3:** Pharmacokinetic and drug-likeness properties of the top five compounds with the highest docking scores against the AmpC protein.

Compounds	TPSA Å^2^	ESOL Class	iLOGP	C-LogP	GI-A	LV	PA	BAS	SA
N094-0017	55.12	Very soluble	1.67	0.94	High	0	0	0.55	2.75
0115-0006	63.6	Very soluble	1.92	1.44	High	0	0	0.55	4.6
N002-0028	72.83	Soluble	2.67	1.91	High	0	0	0.55	4.91
N003-0017	63.6	Soluble	1.92	1.68	High	0	0	0.55	4.16
0080-0056	59.67	Soluble	2.46	2.13	High	0	0	0.55	3.55

The parameters include TPSA (Topological Polar Surface Area, Å^2^), ESOL Class (aqueous solubility prediction), iLOGP (predicted lipophilicity), C-LogP (consensus LogP value), GI-A (Gastrointestinal Absorption), LV (Lipinski Rule of Five violations), PA (PAINS alerts), BAS (Bioavailability Score), and SA (Synthetic Accessibility score).

### 3.3 Molecular dynamics and simulation

The stability and structural behavior of AmpC protein-ligand complexes were investigated using MD simulations. This analysis evaluates the strength, stability, interactions, and dynamic aspects of protein-ligand binding. Furthermore, MD simulations provide vital insights into how macromolecules change shape in aquatic biological systems. The stability, folding behavior, and dynamic interactions of the protein-ligand complexes were assessed during the simulation using a variety of trajectory parameters, including hydrogen-bond analysis, RMSD, and RMSF. The AmpC-ligand combination and the AmpC protein were both simulated for 500 ns. To determine the key structural and energetic components influencing binding stability, we conducted a thorough analysis of the protein’s dynamics in both the free and bound states.

#### 3.3.1 RMSD analysis

**FIGURE 2:**
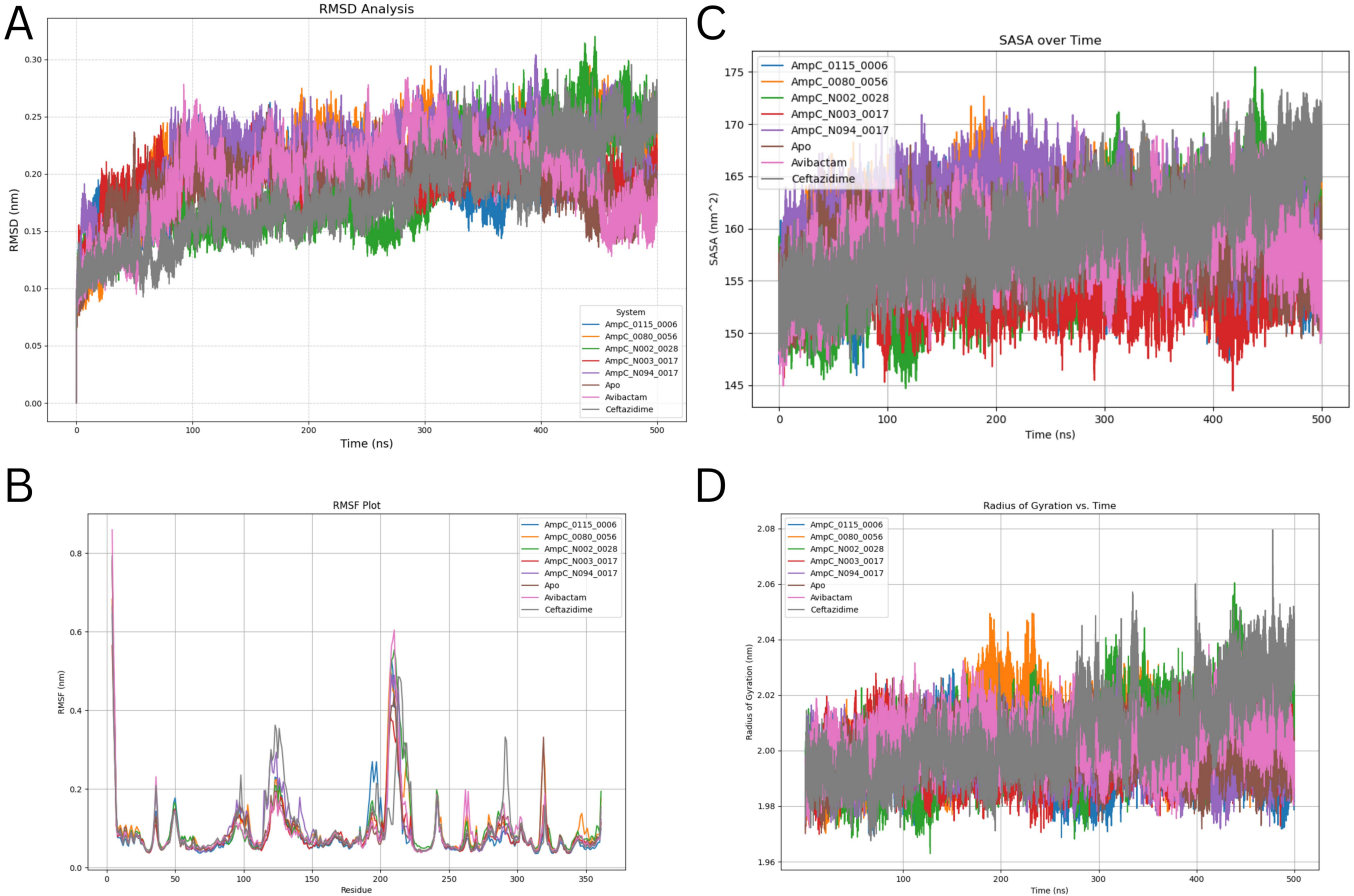
Displays the MD analysis, which includes: **(A)** Root Mean Square Deviation (RMSD), **(B)** Root Mean Square Fluctuation (RMSF), **(C)** Solvent Accessible Surface Area (SASA), and **(D)** Radius of Gyration (RG). The plots are based on a 500 ns MD simulation.

RMSD quantifies the degree to which a protein’s structure deviates from a reference conformation over time, serving as a reliable indicator of structural stability throughout the simulation. The RMSD profiles for all AmpC complexes over the 500 ns trajectory are shown in Figure 2A, highlighting clear differences in stability across the ligand-bound systems. The apo form of AmpC maintained a consistently low RMSD of 0.097 ± 0.011 nm, reflecting strong structural rigidity in the absence of any bound ligand. Modest increases were noted upon ligand binding, with the Avibactam (0.107 ± 0.008 nm) and Ceftazidime (0.107 ± 0.013 nm) complexes showing minor conformational adjustments in response to ligand interaction. Among the ChemDiv compounds, AmpC_0080-0056 showed high stability with an RMSD of 0.085 ± 0.0095 nm, closely mirroring the apo structure. The AmpC_N002_0028 and AmpC_N003_0017 complexes recorded moderately higher values of 0.117 ± 0.015 nm and 0.118 ± 0.0152 nm, respectively, indicating moderate structural flexibility upon binding. The AmpC_0115_0006 complex behaved similarly, with an RMSD of 0.116 ± 0.0171 nm. The AmpC_N094_0017 complex exhibited the highest RMSD of 0.1393 ± 0.0303 nm, reflecting greater conformational fluctuations during the run. Despite this, values remained within an acceptable range, confirming stable ligand accommodation within the active site. Overall, compounds 0080-0056 and N094-0017 displayed the most favorable stability profiles and were selected as promising candidates for further dynamic and free energy analyses.

#### 3.3.2 RMSF analysis

Individual residue flexibility is measured by RMSF, which shows how much each residue moves throughout a simulation. The RMSF for each residue is typically plotted against the residue number to identify the amino acids that contribute most to the molecular motion in a protein (Figure 2B). The average RMSF for the apo form was 0.089 ± 0.07 nm, which suggests that the structure is somewhat flexible overall. Ligand binding resulted in changes in residue-level flexibility, reflecting localized structural adaptations upon complex formation. The Avibactam and Ceftazidime complexes showed slightly increased RMSF values of 0.093 ± 0.086 nm and 0.099 ± 0.081 nm, respectively, suggesting modest increases in flexibility relative to the apo form, likely due to localized interactions within the active site. Among the ChemDiv-derived complexes, AmpC_0115-0006 and AmpC_N003-0017 exhibited reduced residue fluctuations with average RMSF values of 0.085 ± 0.077 nm and 0.0849 ± 0.0614 nm, respectively, indicating enhanced structural stability in key functional regions. In contrast, the AmpC_0080-0056 and AmpC_N002-0028 complexes showed comparatively higher flexibility, with RMSF values of 0.098 ± 0.077 nm and 0.0993 ± 0.088 nm, respectively. The AmpC_N094-0017 complex displayed an intermediate RMSF value of 0.0939 ± 0.084 nm, suggesting balanced flexibility while maintaining stable ligand interactions. Overall, the RMSF analysis demonstrates that ligand binding modulates residue-level dynamics of AmpC β-lactamase, with compounds 0115-0006 and N003-0017 conferring enhanced rigidity, while N094-0017 maintains favorable dynamic stability suitable for effective binding.

#### 3.3.3 SASA analysis

We calculated the SASA, which measures the protein’s surface area accessible to solvent molecules such as water. SASA is an important parameter in protein structure analysis, providing insights into protein folding, stability, and interactions with other molecules. The SASA analysis indicates the degree of protein surface exposure to the solvent, highlighting structural changes and the impact of ligand binding (Figure 2C). The apo form of AmpC exhibited a SASA value of 158.08 ± 2.564 nm^2^, representing the baseline solvent exposure of the unbound protein. Upon binding of single reference ligands, slight increases in SASA were observed for the Avibactam (158.52 ± 3.148 nm^2^) and Ceftazidime (159.58 ± 4.38 nm^2^) complexes, suggesting minor surface expansion following ligand interaction. Among the ChemDiv-derived complexes, AmpC_N094-0017 displayed the highest SASA value (161.14 ± 2.957 nm^2^), indicating increased solvent exposure and potential conformational expansion. Similarly, the AmpC_0080-0056 complex showed a relatively elevated SASA value of 160.0 ± 2.879 nm^2^. In contrast, the AmpC_N003-0017 complex exhibited a reduced SASA value of 155.10 ± 2.60 nm^2^, suggesting a more compact protein conformation. The AmpC_0115-0006 and AmpC_N002-0028 complexes demonstrated moderate SASA values of 157.23 ± 2.842 nm^2^ and 157.62 ± 4.357 nm^2^, respectively, comparable to the apo structure. Overall, the SASA analysis indicates that ligand binding induces compound-specific effects on protein surface exposure. While compounds AmpC_N094-0017 and AmpC_0080-0056 increased solvent accessibility, AmpC_N003-0017 promoted a more compact protein conformation, suggesting enhanced structural stability. These findings highlight the influence of ligand binding on the conformational dynamics of AmpC β-lactamase and support further evaluation of ChemDiv-derived compounds.

#### 3.3.4 Rg analysis

We conducted a Rg analysis to evaluate the overall size and compactness of the protein molecule. Rg provides information on protein’s kinetics and structural characteristics by measuring the mass distribution around their center of mass. The Rg values for the AmpC protein and its ligand-bound complexes were analyzed to assess changes in structural compactness upon ligand binding (Figure 2D). The apo form of AmpC (Apo) exhibited an average Rg of 1.99 ± 0.0064 nm, indicating a stable and compact structure. In the single-ligand complexes, Avibactam and Ceftazidime showed slightly increased Rg values of 2.003 ± 0.0080 nm and 2.005 ± 0.0145 nm, respectively, suggesting minor structural expansion upon ligand binding. Among the ChemDiv-derived ligand complexes, AmpC_0115_0006 and AmpC_N094_0017 demonstrated relatively compact and stable conformations with Rg values of 1.996 ± 0.00831 nm and 1.996 ± 0.0069 nm, respectively. In contrast, AmpC_0080_0056 and AmpC_N002_0028 displayed modest structural expansion, reflected by higher Rg values of 2.004 ± 0.0109 nm and 2.002 ± 0.0129 nm, respectively. Interestingly, AmpC_N003_0017 maintained a compact structure with an Rg value of 1.99 ± 0.00734 nm, comparable to the apo form. Overall, these results indicate that although ligand binding induces minor variations in structural compactness, select ChemDiv ligands can maintain the structural integrity and stability of the AmpC protein.

#### 3.3.5 H bond analysis

**FIGURE 3:**
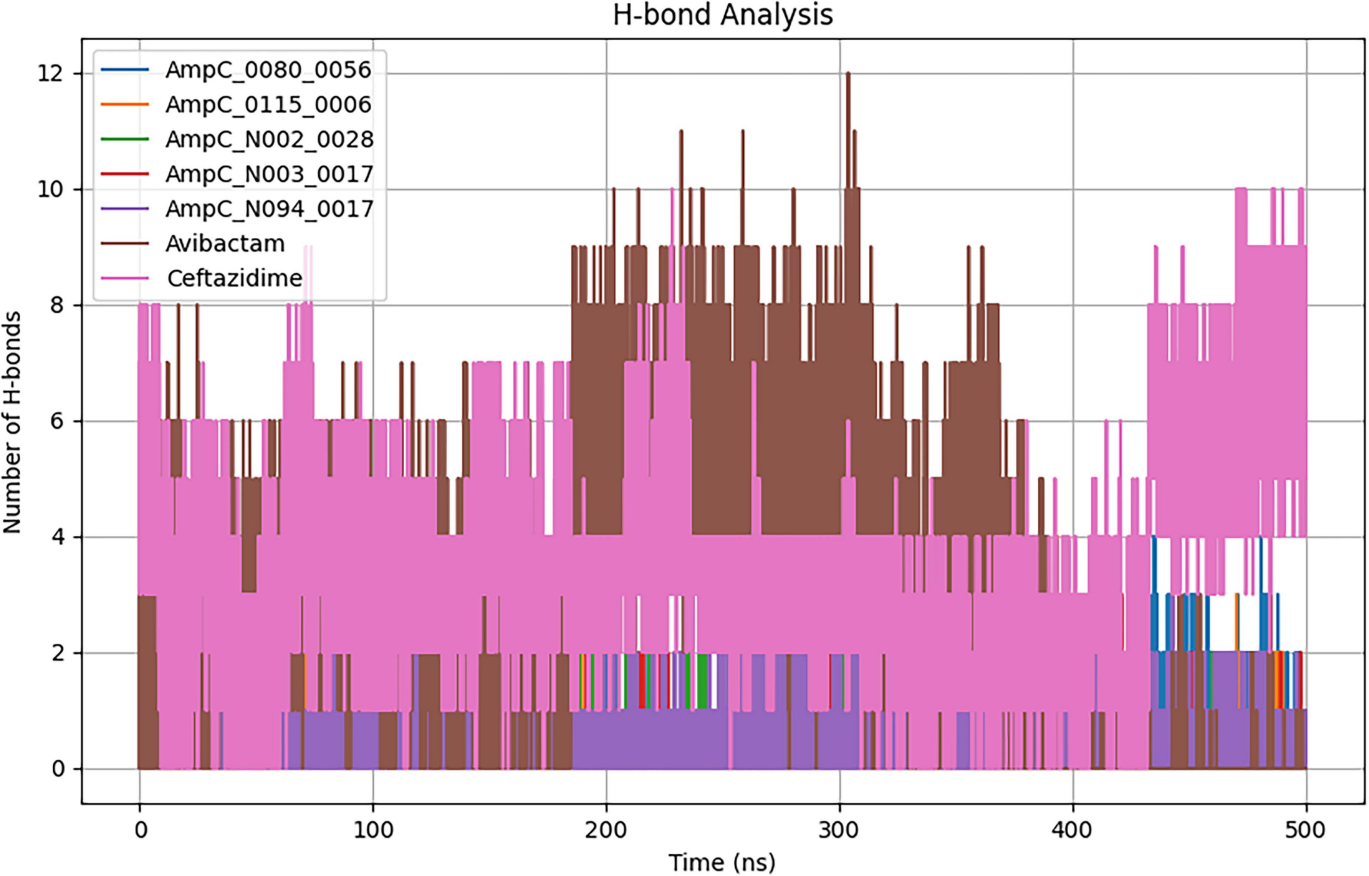
Quantifies hydrogen bond formations observed throughout the 500 ns MD simulation.

Hydrogen-bond studies revealed that the AmpCligand complexes exhibit varying interaction strengths (Figure 3). Ceftazidime created the most hydrogen bonds (4), indicating the strongest contact. Avibactam and AmpC_0080_0056 each formed two bonds, indicating moderate interaction. AmpC_0115_0006, AmpC_N002_0028, and AmpC_N003_0017 all established three bonds, indicating stable binding. AmpC_N094_0017 produced two hydrogen bonds, indicating fewer bonds than the other complexes.

#### 3.3.6 PCA

**FIGURE 4:**
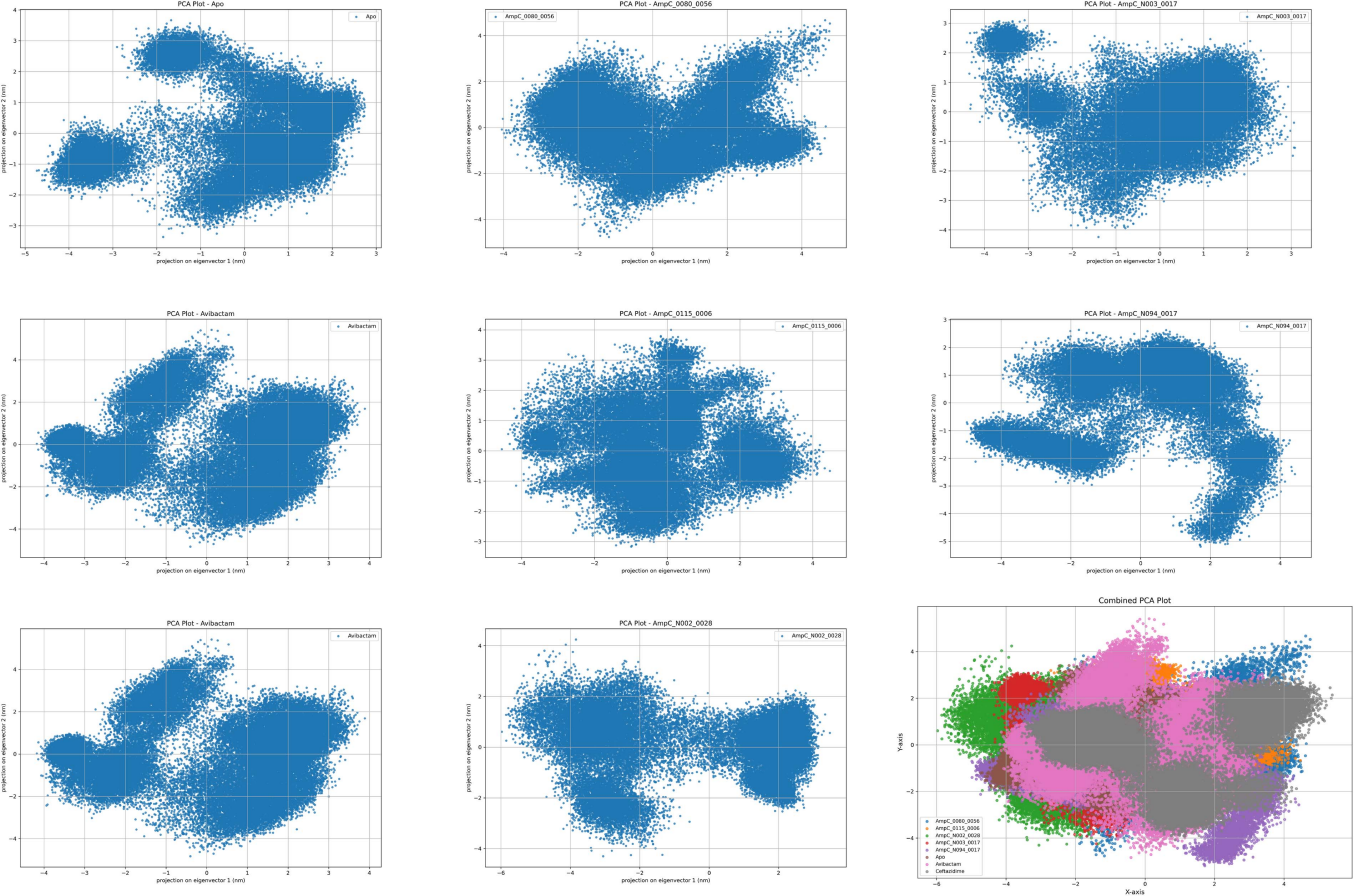
Displays the principal component analysis (PCA) plot illustrating the conformational clustering of protein-ligand complexes throughout the MD simulation.

The PCA results reveal details on the stability and conformational flexibility of the AmpC complexes based on clustering patterns in the principal component space (Figure 4). The AmpC_N094-0017 complex exhibited a compact and well-defined cluster, indicating high structural stability and limited conformational fluctuations. The apo system displayed a dense cluster with moderate dispersion, suggesting overall structural stability with inherent flexibility. The avibactam-bound system showed a dense cluster with slight elongation, reflecting moderate stability and controlled conformational variation. In contrast, the ceftazidime-bound system exhibited multiple distinct clusters, indicating increased conformational heterogeneity and reduced stability relative to the other complexes. Among the ChemDiv-derived ligands, the AmpC_0115-0006 complex formed a relatively dense cluster with mild elongation, suggesting moderate variability. The AmpC_0080-0056 complex showed a more dispersed clustering pattern, indicative of higher conformational flexibility and reduced stability. The AmpC_N003-0017 complex demonstrated intermediate dispersion, reflecting balanced stability and flexibility, whereas the AmpC_N002-0028 complex displayed the most dispersed distribution, suggesting the lowest structural stability among all analyzed systems.

#### 3.3.7 FEL

**FIGURE 5:**
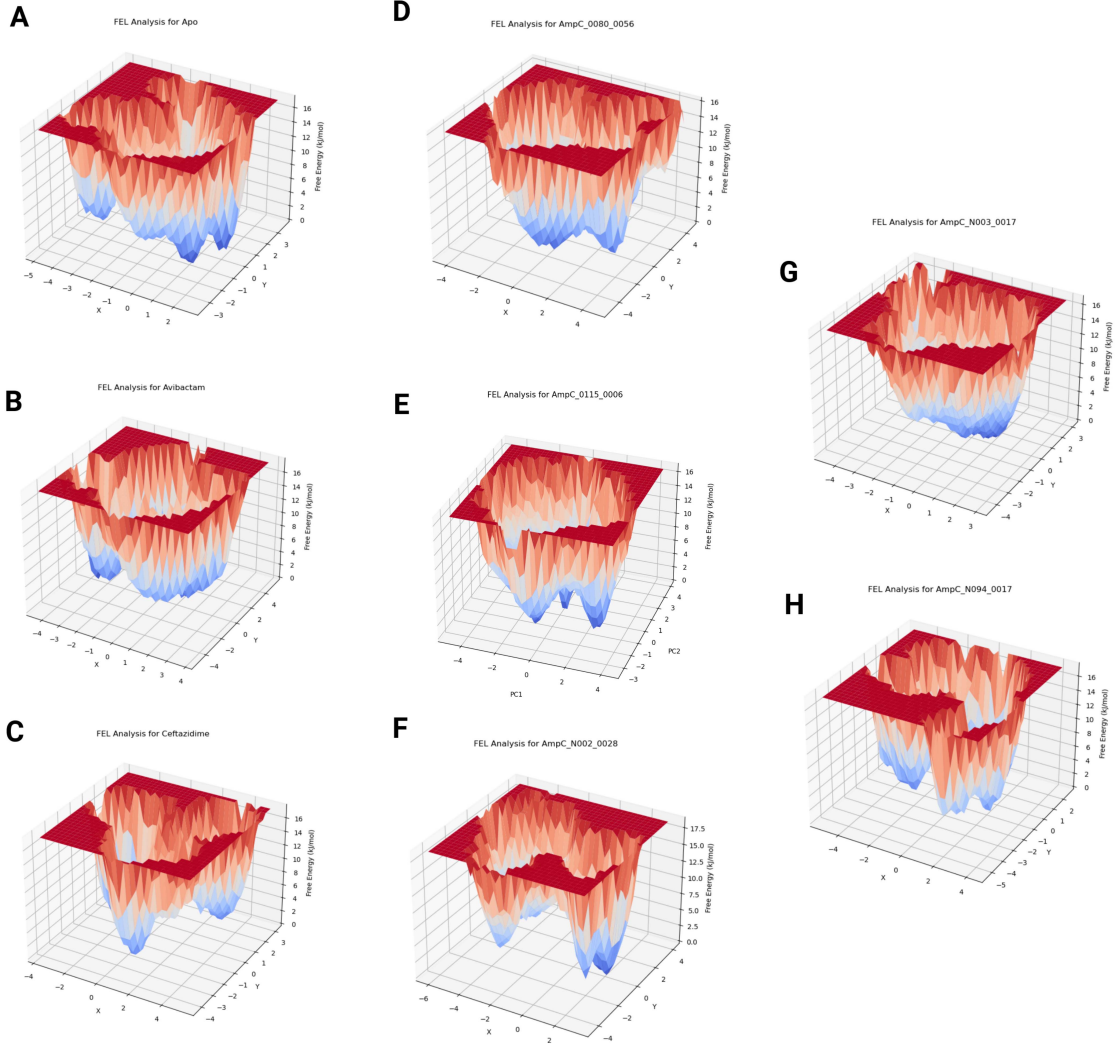
Presents the free energy landscape (FEL) analysis, showing the structural conformations of the protein-ligand complex throughout the molecular dynamics MD simulation. **(A)** Apo, **(B)** Avibactam, **(C)** Ceftazidime, **(D)** 0080-0056, **(E)** 0115-0006, **(F)** N002-0028, **(G)** N003-0017, **(H)** N094-0017.

The free energy landscape (FEL) analysis (Figure 5) reveals the conformational stability and dynamic behavior of AmpC in apo and ligand-bound states by mapping the thermodynamically favorable conformations sampled during the MD simulations. Among all complexes, the AmpC_N094-0017 system exhibited the highest conformational stability, characterized by a single, deep, and well-defined free-energy minimum, indicating a dominant and stable bound conformation with reduced conformational heterogeneity. This narrow and deep energy basin suggests effective conformational locking of the protein upon ligand binding. The apo, avibactam-bound, and ceftazidime-bound systems displayed multiple low-energy minima with broader basins, reflecting increased conformational flexibility and the presence of several metastable states rather than a single dominant conformation. Such behavior is indicative of dynamic binding and active-site plasticity. In contrast, the AmpC_0080-0056 and AmpC_0115-0006 complexes exhibited shallow, fragmented energy basins, indicating weak stabilization, whereas the AmpC_N002-0028 and AmpC_N003-0017 systems displayed poorly defined minima, reflecting greater structural fluctuations. Overall, the FEL results are consistent with the PCA analysis, demonstrating that ligands that induce deep, well-defined free-energy minima stabilize AmpC conformations, whereas weaker binders permit greater protein flexibility.

#### 3.3.8 Dynamic cross correlation matrix (DCCM)

**FIGURE 6:**
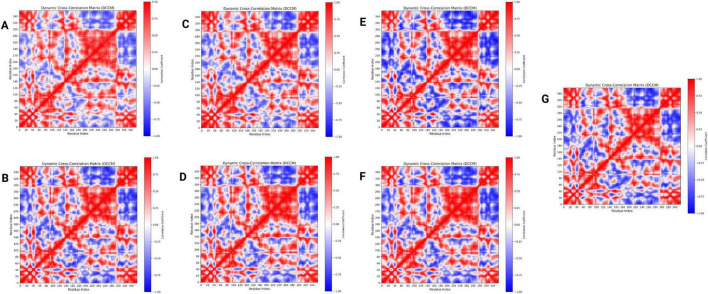
DCCM analysis of the ligand-bound AmpC complex. **(A)** Avibactam, **(B)** Ceftazidime, **(C)** 0080-0056, **(D)** 0115-0006, **(E)** N002-0028, **(F)** N003-0017, **(G)** N094-0017.

DCCM analysis was used to evaluate how ligand binding affected the linked movements of AmpC protein residues (Figure 6). The avibactam (A) and ceftazidime-bound (B) complexes exhibit extensive regions of strong positive correlation (correlation coefficient > 0.5), particularly across residues 60–80 and 150–170, accompanied by a marked reduction in anti-correlated motions (correlation coefficient < −0.3). This pattern indicates enhanced coordination of collective residue movements and partial restriction of opposing fluctuations within these regions. In contrast, the complexes formed with the ChemDiv compounds, 0080-0056 (C), 0115-0006 (D), N002-0028 (E), N003-0017 (F), and N094-0017 (G), display more fragmented positive correlation patterns with persistent anti-correlated off-diagonal regions, suggesting incomplete coupling of protein motions and retention of dynamic flexibility. Based on the extent and continuity of correlated residue motions, avibactam and ceftazidime exhibit the strongest dynamic stabilization, followed by 0080-0056 and N094-0017, while 0115-0006, N002-0028, and N003-0017 show comparatively weaker coupling. Overall, avibactam and ceftazidime serve as reference, underscoring the importance of promoting long-range motion coupling when optimizing novel AmpC inhibitors for studies of antimicrobial resistance.

#### 3.3.9 MM-PBSA

The MM-PBSA analysis Table 4 (Supplementary Figure 3) identifies Ceftazidime as the strongest candidate, with the highest binding energy of −238.457 ± 35.980 kJ/mol, indicating superior binding affinity. However, the large positive polar solvation energy (662.254 kJ/mol) reflects a substantial desolvation penalty, which may influence its overall stability in the binding environment. Among the screened compounds, N094_0017 demonstrated comparatively moderate binding energy (−35.171 ± 8.823 kJ/mol) with low standard deviation, suggesting stable and consistent interactions within the active site. In contrast, other candidates such as N002_0028 and 0080_0056 showed weaker binding energies accompanied by higher fluctuations, indicating less stable interactions. Overall, while Ceftazidime remains the strongest binder according to MM-PBSA analysis, N094_0017 appears as a promising novel candidate with stable binding properties that requires further experimental confirmation. It is crucial to note that MM-PBSA produces relative binding free-energy estimations, which may not be directly comparable to absolute free-energy values obtained by alchemical FEP calculations.

**TABLE 4:** MM-PBSA binding free energy analysis of the selected protein–ligand complexes targeting the AmpC protein.

Protien-ligand complex	van der Waal energy (kJ/mol)	Electrostattic energy (kJ/mol)	Polar solvation energy (kJ/mol)	SASA energy (kJ/mol)	Binding energy (kJ/mol)
Avibactam	−0.601	−54.384	13.707	−0.032	−41.311
Ceftazidime	−82.074	−803.508	662.254	−15.129	−238.457
0080_0056	−49.256	−6.86	48.539	−6.763	−14.339
0115_0006	−7.351	−7.091	41.761	−1.353	25.966
N002_0028	−84.812	−15.883	71.85	−11.585	−40.43
N003_0017	−13.191	−0.311	−5.021	−2.191	−20.714
N094_0017	−126.146	−17.888	121.945	−13.083	−35.171

The energy contributions include van der Waals energy, electrostatic energy, polar solvation energy, and SASA (Solvent Accessible Surface Area) energy, with the final binding free energy (kJ/mol) calculated as the sum of these components.

### 3.10 Alchemical free energy perturbation (FEP) analysis

**FIGURE 7:**
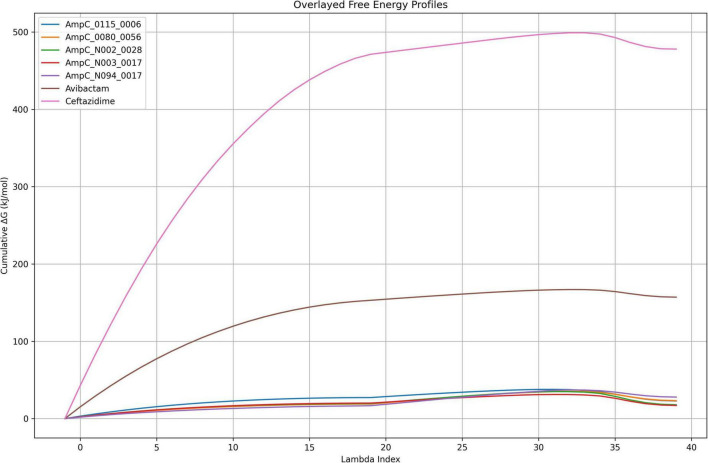
Free energy perturbation (FEP) profiles of AmpC–ligand complexes along the λ coordinate, illustrating relative binding free energy changes and energetic stability across the transformation.

Alchemical free energy perturbation (FEP) calculations were performed to quantitatively estimate the binding free energies of selected ligands to AmpC β-lactamase (Figure 7). All systems exhibited smooth and continuous ΔG progression, indicating well-converged simulations and stable alchemical transformations. The cumulative free energy profiles revealed a clear separation between reference inhibitors and ChemDiv-derived compounds. Among all ligands, Ceftazidime showed the highest cumulative ΔG, reaching approximately 480–500 kJ/mol, indicating the strongest binding affinity, as a greater amount of energy is required to decouple the ligand from the protein environment. Avibactam displayed intermediate cumulative ΔG values (∼155–165 kJ/mol), reflecting moderate binding affinity and consistent convergence behavior. In contrast, all ChemDiv compounds exhibited substantially lower cumulative ΔG values, generally within the range of ∼20–30 kJ/mol, indicating comparatively weaker absolute binding affinities. Among these, N094-0017 showed a stable, gradual increase in ΔG to ∼40 kJ/mol, suggesting consistent interaction with the binding site. Similarly, compounds 0115-0006 and 0080-0056 demonstrated comparable profiles with slightly higher terminal ΔG values (∼20–35 kJ/mol), indicating moderate binding behavior. N003-0017 and N002-0028 exhibited lower cumulative ΔG values (∼20–30 kJ/mol) with minor fluctuations, suggesting relatively weaker and less stable interactions compared to the top-performing screened compounds. Overall, the FEP profiles clearly demonstrate that while reference drugs such as Ceftazidime exhibit significantly stronger binding, the screened ChemDiv compounds, particularly N094-0017, maintain stable interaction patterns and represent promising lead candidates for further optimization.

## 4 Discussion

The rapid increase in AMR, especially in MDR pathogens like *P. aeruginosa*, poses a major global health threat (Ahmed et al., 2024). A significant factor in AMR for *P. aeruginosa* is the enzyme AmpC β-lactamase, which breaks down β-lactam antibiotics, making them ineffective. Encoded by the chromosomal blaAmpC gene, this enzyme exhibits natural resistance to various β-lactam antibiotics, including monobactams, cephalosporins, and aminopenicillins (Upadhyay et al., 2013). Mutations in the peptidoglycan recycling mechanism often led to the overproduction of AmpC, which is commonly associated with resistance to antipseudomonal drugs like cephalosporins, ticarcillin, and piperacillin. Extended-spectrum AmpC (ESAC) variants and structural changes further enhance this resistance (Upadhyay et al., 2013; Berrazeg et al., 2015). AmpC overproduction is also linked to biofilm formation, significantly contributing to antibiotic resistance by limiting drug penetration and enhancing efflux mechanisms. Bacteria within biofilms exhibit much higher minimum inhibitory concentrations (MICs) than planktonic cells, complicating treatment (Tuon et al., 2022). Although β-lactam/β-lactamase inhibitor (BLBLI) combinations such as ceftazidime/avibactam have shown considerable promise against AmpC-producing strains, their clinical effectiveness is frequently undermined by overlapping resistance mechanisms, most notably AmpC overproduction, loss of outer membrane porins, and upregulation of efflux pumps (Ferous et al., 2024). The therapeutic landscape for BLBLI-based treatment has nonetheless continued to evolve, with several newer combinations gaining clinical approval in recent years. Agents such as cefepime/enmetazobactam, aztreonam/avibactam, and sulbactam/durlobactam have entered clinical use and represent meaningful additions to the available options for managing difficult-to-treat infections (Sargianou et al., 2025; Keam, 2024). Within this expanding armamentarium, cefepime/enmetazobactam is primarily directed against extended-spectrum β-lactamase (ESBL)-producing *P. aeruginosa* and Enterobacterales, while aztreonam/avibactam broadens coverage to include carbapenem-resistant Enterobacterales harboring metallo-β-lactamases (Carcione et al., 2026). Despite these developments, existing BLBLI combinations remain susceptible to emerging resistance mechanisms, particularly AmpC overproduction, porin mutations, and efflux pump activation. A further limitation is the inadequate coverage of isolates carrying multiple co-occurring resistance determinants, which are increasingly encountered in difficult-to-treat *P. aeruginosa* infections (Karaiskos et al., 2019). The restricted activity of current combinations against metallo-β-lactamases and biofilm-associated infections adds to the growing recognition that alternative therapeutic strategies are urgently needed (Baniya et al., 2017). Against this backdrop, computational approaches including molecular docking, molecular dynamics (MD) simulations, and virtual screening have gained considerable traction as bioinformatics-driven tools for tackling AmpC-mediated resistance. These methods support the rational identification, design, and refinement of novel AmpC β-lactamase inhibitors, offering a systematic and resource-efficient pathway toward the discovery of new therapeutic candidates (Hafeez et al., 2024).

This study identified potential inhibitors of AmpC β-lactamase through virtual screening of 374 compounds from the ChemDiv database. The compounds were docked into the AmpC enzyme’s active site using the Glide docking module, and their binding affinities and interaction characteristics, including hydrogen bonding, were assessed to identify promising candidates. N094-0017 had the best docking score (−5.6 kcal/mol) and created stable hydrogen-bonding contacts with critical active-site residues. However, it is important to note that docking scores represent approximate estimates of binding affinity and are primarily useful for relative ranking within a screened dataset rather than as absolute indicators of inhibitory potency. Compared with reported docking ranges for known AmpC inhibitors, including clinically relevant compounds such as avibactam, this value falls within a moderate binding-affinity range and should therefore be interpreted with caution. Accordingly, the observed docking score highlights the prioritization of N094-0017 among the screened compounds rather than indicating superior inhibitory activity relative to established inhibitors. Comparatively, other compounds in the dataset showed weaker binding affinities and less stable interaction profiles. For instance, compounds such as N153-0023 and N202-0145 demonstrated moderate docking scores of −4.8 and −4.6 kcal/mol, respectively, but lacked significant hydrogen bonding interactions with key catalytic residues, which may reduce their binding stability. In addition to binding characteristics, the pharmacokinetic properties of the top compounds were evaluated using SwissADME. The results indicated that N094-0017 possesses favorable drug-like attributes, including high solubility, moderate lipophilicity, and strong potential for gastrointestinal absorption. While these properties support its suitability as a lead candidate for further development, it is important to emphasize that such predictions are based on computational models and do not directly translate to in vivo efficacy. Overall, N094-0017 demonstrates a balanced profile in terms of binding behavior and predicted pharmacokinetics, supporting its selection for further investigation and experimental validation.

MD simulations were conducted to assess the stability and dynamic behavior of selected inhibitors in complex with AmpC β-lactamase. Among the examined compounds, the N094-0017-AmpC complex demonstrated greater stability, as seen by consistently low RMSD, RMSF, Rg, and SASA values. These metrics indicate minimal structural perturbations, reduced atomic fluctuations, and effective burial of the ligand within the binding pocket. The radius of gyration (Rg) analysis further confirmed that the N094-0017–AmpC complex remained compact throughout the 500 ns simulation, suggesting that ligand binding did not destabilize the overall protein conformation. In contrast, other promising candidates, such as 0080-0056, displayed higher SASA and fluctuating Rg values, indicating weaker binding interactions and less structural compactness. Hydrogen bond analysis revealed that N094-0017 consistently formed stable interactions with key active-site residues, whereas other compounds showed fewer or less persistent bonds, further supporting N094-0017’s robust inhibitory potential. All simulations, including the apo protein and each ligand-bound complex, were conducted under identical conditions for 500 ns. Trajectory convergence was assessed by monitoring RMSD values, which stabilized after an initial equilibration phase, and by observing low standard deviations across RMSD, RMSF, SASA, and Rg, confirming structural stability. These statistical measures, reported as average ± standard deviation over the entire trajectory, provide a quantitative assessment of conformational stability. The MD results further suggest that ligand binding may induce localized conformational adaptations within the AmpC active site. Although most complexes exhibited low RMSD values indicative of structural stability, the slightly higher RMSD observed for the AmpC_N094-0017 complex may reflect adaptive structural rearrangements that facilitate ligand accommodation within the catalytic pocket. Such behavior is consistent with an induced-fit mechanism, where proteins undergo subtle conformational adjustments upon ligand binding to optimize intermolecular interactions. Importantly, despite these fluctuations, the RMSD values remained within a narrow range (<0.15 nm), indicating that the overall structural integrity of the AmpC enzyme was preserved throughout the simulation. These findings suggest that the observed flexibility may contribute to effective ligand binding rather than indicating structural instability. The MM-PBSA method’s binding free energy calculations confirmed these findings, with Ceftazidime exhibiting the most favorable binding free energy among the evaluated compounds, while N094-0017 showed stable and consistent binding among the screened compounds. The energy component decomposition revealed that van der Waals and electrostatic interactions are crucial in stabilizing the AmpC_N094-0017 complex. Compared to other candidates, N094-0017 showed improved stability across multiple parameters. While compounds such as N002-0028 displayed moderate behavior, others including N153-0023 and 0115-0006 exhibited weaker interaction profiles, further highlighting N094-0017 as a promising inhibitor among the screened compounds. It is important to note that MM-PBSA and FEP results are not directly comparable, as they represent relative binding energies and absolute decoupling free energies, respectively. To provide a rigorous thermodynamic validation of ligand binding, FEP calculations were performed. The cumulative free energy profiles revealed distinct differences in binding energetics between reference inhibitors and ChemDiv-derived compounds. Ceftazidime exhibited a steep increase in cumulative free energy (∼480–500 kJ/mol), indicating higher binding affinity. This is because higher cumulative ΔG values correspond to greater energy required to decouple the ligand from the binding site, likely due to significant desolvation penalties associated with its large size and high polarity. Avibactam exhibited intermediate cumulative ΔG values (155–165 kJ/mol), indicating moderate binding affinity compared to Ceftazidime. In contrast, the ChemDiv compounds, including N094-0017, exhibited lower cumulative ΔG values, indicating comparatively weaker absolute binding affinities, which may be attributed to their smaller size and reduced polarity, resulting in fewer extensive electrostatic and desolvation interactions compared to reference inhibitors. the Ceftazidime–Avibactam combination therapy faces challenges due to emerging bacterial resistance mechanisms, which can neutralize both the β-lactam antibiotic and its inhibitor (Zasowski et al., 2015). Overall, these findings identify N094-0017 as a promising lead compound with stable binding behavior, suitable for further optimization rather than serving as a direct replacement for existing inhibitors in combating β-lactam resistance. Furthermore, *in vitro* validation must be conducted to confirm their inhibitory and cytotoxic activities. Moreover, *in-vivo* studies are required to evaluate its pharmacokinetics, efficacy, and safety in a physiological environment.

## 5 Conclusion

This study puts forward N094-0017 as a potential inhibitor of AmpC β-lactamase, supported by a stable interaction profile and favorable binding characteristics observed across molecular docking and molecular dynamics simulations. The compound also demonstrated a desirable pharmacokinetic profile, further supporting its candidacy as a lead molecule against AmpC-producing *P. aeruginosa*. Although reference compounds such as ceftazidime and avibactam display stronger absolute binding affinities, N094-0017 maintained consistent stability and well-defined interaction behavior throughout the simulations, underscoring its suitability as a foundation for further structural optimization. Rather than functioning as a direct replacement for existing treatments, N094-0017 is better regarded as a potential alternative scaffold upon which next-generation inhibitors could be developed, with the aim of complementing therapies such as ceftazidime–avibactam that are increasingly challenged by evolving resistance mechanisms. The findings presented here lay the groundwork for subsequent experimental validation, encompassing *in vitro* assays to confirm inhibitory activity, cytotoxicity profiling, and comparative efficacy studies against currently available standard treatments. Future work should also investigate the compound’s stability under physiological conditions and explore whether synergistic effects can be achieved when combined with existing antibiotics. Broadly, this study positions N094-0017 as a promising lead compound for rational drug design, contributing meaningfully to the ongoing pursuit of new therapeutic strategies to tackle antimicrobial resistance and broaden the available options for managing infections caused by resistant bacterial pathogens.

## Data Availability

The original contributions presented in this study are included in the article/Supplementary material, further inquiries can be directed to the corresponding authors.
