# Supplementary material for: Identification of a novel natural compound inhibitor targeting AmpC β-lactamase to combat multidrug-resistant Pseudomonas aeruginosa
Source: Front Microbiol. 2026 May 19;17:1813201. doi: 10.3389/fmicb.2026.1813201 (PMC13226586; doi:10.3389/fmicb.2026.1813201)
Supplement: Supplementary file 1 [file Table_1.docx]

**Supplementary Material:**


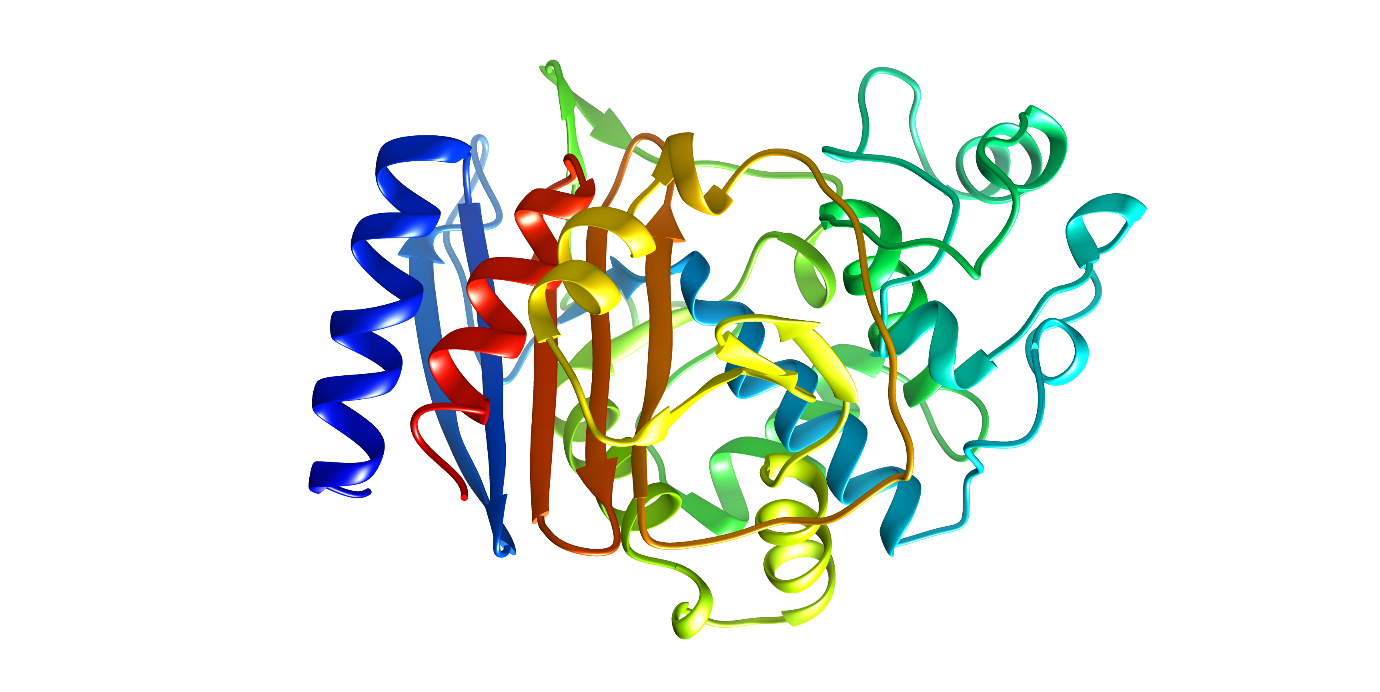


**Supplementary Figure 1:** 3D Structure of the AmpC


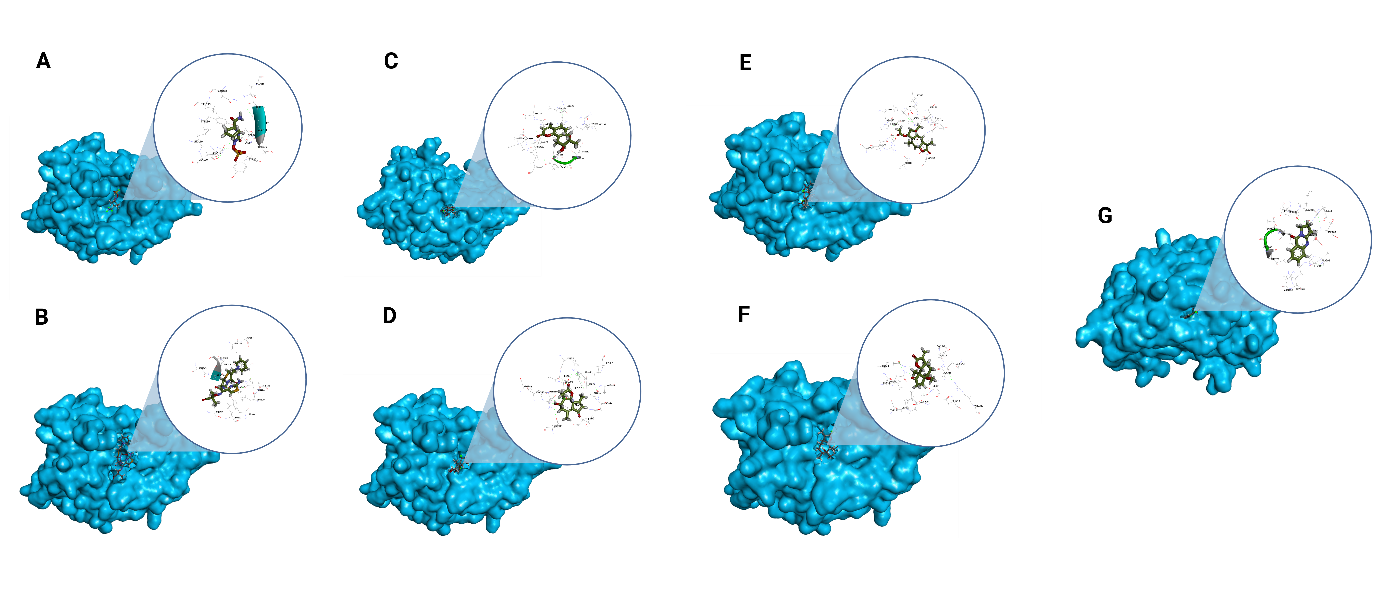


**Supplementary Figure 2:** 3D Interaction of the AmpC and the compounds.


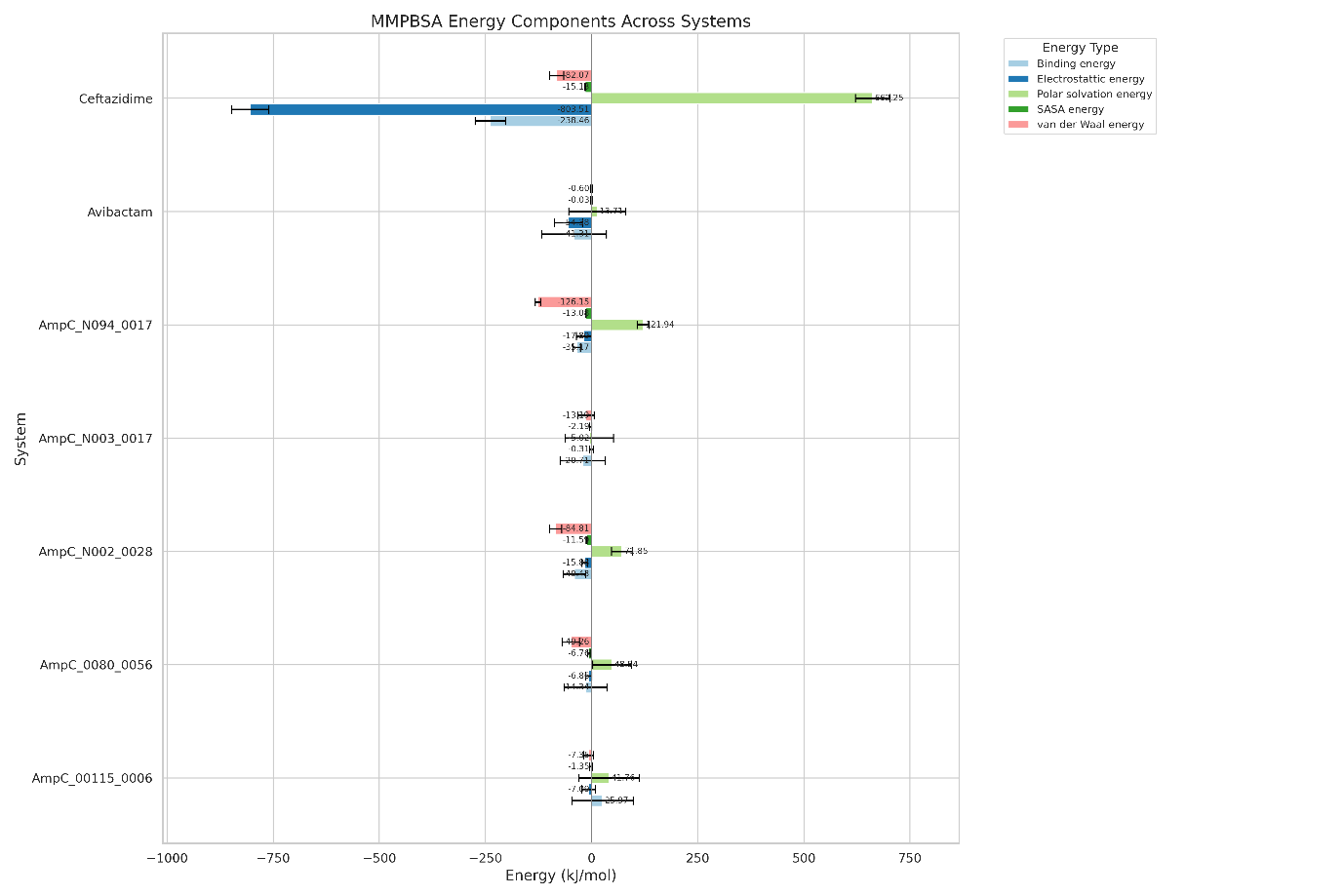


**Supplementary Figure 3:** Overview of MMPBSA Analysis
